# Physicochemical Properties of Nanomaterials: Implication in Associated Toxic Manifestations

**DOI:** 10.1155/2014/498420

**Published:** 2014-08-06

**Authors:** Manzoor Ahmad Gatoo, Sufia Naseem, Mir Yasir Arfat, Ayaz Mahmood Dar, Khusro Qasim, Swaleha Zubair

**Affiliations:** ^1^Department of Biochemistry, Jawaharlal Nehru Medical College, Aligarh Muslim University, Aligarh, Uttar Pradesh 202002, India; ^2^Department of Chemistry, Aligarh Muslim University, Aligarh, Uttar Pradesh 202002, India; ^3^Department of Mechanical Engineering, Aligarh Muslim University, Aligarh, Uttar Pradesh 202002, India; ^4^Women's College, Aligarh Muslim University, Aligarh, Uttar Pradesh 202002, India

## Abstract

Nanotechnology has emerged as one of the leading fields of the science having tremendous application in diverse disciplines. As nanomaterials are increasingly becoming part of everyday consumer products, it is imperative to assess their impact on living organisms and on the environment. Physicochemical characteristics of nanoparticles and engineered nanomaterials including size, shape, chemical composition, physiochemical stability, crystal structure, surface area, surface energy, and surface roughness generally influence the toxic manifestations of these nanomaterials. This compels the research fraternity to evaluate the role of these properties in determining associated toxicity issues. Reckoning with this fact, in this paper, issues pertaining to the physicochemical properties of nanomaterials as it relates to the toxicity of the nanomaterials are discussed.

## 1. Introduction

Nanotechnology is being considered as the next step logical in integrating technology based science with other sister disciplines including biology, chemistry, and physics [1]. Royal Society and Royal Academy of Engineering have defined “nanoscience” as the study of phenomena and manipulation of materials at atomic, molecular, and macromolecular scales while nanotechnology has been defined as the design, characterization, production, and application of structures, devices and systems by controlling shape and size at nanometre scale [2]. Current nanotechnology is the building device of microscopic or even molecular size, which will potentially be benefiting medicine, environmental protection, energy, and space exploration [3–6]. In the last few years, the term “nanotechnology” has been inflated and has almost become synonymous for objects that are innovative and highly promising [5, 7–9]. A more generalized description of nanotechnology could be manipulation of matter with at least one dimension of size from 1 to 100 nanometres, namely, nanomaterials. Intriguingly, these nanomaterials embody distinctive physicochemical and biological properties compared to their conventional counter parts which endow them their beneficial characteristics.

In the recent scenario, researches engrossing different nanoparticles are evolving at a tremendous pace owing to which engineered nanomaterials (ENMs) are increasingly becoming part of daily life in the form of cosmetics, food packaging, drug delivery, therapeutics, biosensors, and so forth and, with these, unprecedented avenues for exposure of nanoparticles (NPs) to environment and living beings are increasing [10]. The increasing exposure of nanomaterials makes it imperative to assess the toxic effect of nanoparticle based materials; moreover, as the physical and chemical characteristics of nanomaterials influence the properties of nanoparticles, it is also more imperative to evaluate the physicochemical properties of nanomaterials including size, surface area, solubility, chemical composition, shape, agglomeration state, crystal structure, surface energy, surface charge, surface morphology, and surface coating and also role of individual characteristic property in imparting toxic manifestations. Reckoning with these facts, in this review, an attempt has been made to analyse the corelation of these physicochemical properties with the toxicity of engineered nanomaterials.

It is in general consensus that nanoparticles exhibit toxic manifestations through diverse mechanisms and can result in allergy, fibrosis, organ failure, nephrotoxicities, haematological toxicities, neurotoxicities, hepatological toxicities, splenic toxicities, and pulmonary toxicities, among others [11–14].

## 2. Physicochemical Properties of Nanoparticles and Their Effect on Toxicity

As a matter of fact, nanomaterials have unique properties relative to bulk counterpart which impart them beneficial characteristics; ironically, they may also bestow them with unique mechanisms of toxicity. In general, toxicity has been thought to originate from nanomaterials' size and surface area, composition, shapes, and so forth as reviewed in the following sections.

### 2.1. Size and Surface Area of the Particles

Particle size and surface area play a major role in interaction of materials with biological system. Seemingly, decreasing the size of the materials leads to an exponential increase in surface area relative to volume, thereby making the nanomaterial surface more reactive on itself and to its contiguous milieu. Of note, particle size and surface area dictate how the system responds to, distributes, and eliminates the materials [15]. It has been established that various biological mechanisms including endocytosis, cellular uptake, and efficiency of particle processing in the endocytic pathway are dependent on size of the material [12, 16]. Various researchers have evaluated* in vitro* cytotoxicity of NPs of different size employing various cell types, culture conditions, and exposure times [17, 18]; however, their* in vivo* evaluation is difficult owing to their more complex nature in the biological systems and requires more comprehensive understanding of the particles [19], though various authors have evaluated their toxicity issues in biological systems employing various* in vivo* models. In general, the size dependent toxicity of nanoparticles can be attributed to its ability to enter into the biological systems [20] and then modify the structure of various macromolecules [21], thereby interfering with critical biological functions.

One of the major mechanisms for* in vivo* toxicity of the ENMs is through the generation of oxidative responses by formation of free radicals, in which size has a decisive role to play as highlighted by many authors that the smaller the size the more able it is towards formation of ROS. These free radicals have been known to impart hazards to biological systems mainly through DNA damage, through oxidation of lipids, and by ensuing of inflammatory responses.

Furthermore, several studies employing diverse class of nanoparticles showed that surface area is also critical factor in displaying toxic manifestations (lung and other epithelial-induced inflammatory responses) in rodents [22]. With decrement in size of nanoparticles, surface area increases which causes a dose dependent increment in oxidation and DNA damaging abilities of these nanomaterials [23] much higher than larger particles with the same mass dose [24].

Nanoparticle size also dictates their pharmacological behaviours. It has been observed that NPs smaller than 50 nm (administrated by intravenous injection) transverse quickly to nearly all tissues and impart potentially toxic manifestations in various tissues; on the other hand, NPs greater than 50 nm (in particular 100–200 nm positively charged particles) are readily taken up by RES which refrain their path to other tissues [25]. Although the clearance by reticuloendothelial system (RES) safeguard other tissues, it makes RES organs such as the liver and spleen as main targets of oxidative stress.

Several toxicological studies have demonstrated that smaller nanoparticles of dimensions <100 nm cause adverse respiratory health effects compared to larger particles of the same material [24, 26]. Inhaled particles of different sizes exhibit different fractional depositions within human respiratory tract. It has been observed that ultrafine particles with diameters <100 nm deposits in all regions, whereas particles <10 nm deposits in the tracheobronchial region, while particles between 10 and 20 nm deposits in the alveolar region [27]. As a result, the translocation or distribution of NPs has been found to be size dependent, which in turn decide their toxicities issues.

Kreyling et al. [28] showed that instillation of Ir192-particles of 80 nm resulted in accumulation in the rat liver with an extent of 0.1% of total amount, while particles of 15 nm size displayed increased accumulation to an extent of 0.3–0.5%. Moreover, it has been observed that when smaller particles are retained in the respiratory tract for longer duration it leads to increased translocation to the pulmonary interstitium with impairment of alveolar macrophages function. Redistribution of NPs from their site of deposition [29] or deposition into renal tissues and escape from normal phagocytic defences [30] may also lead to toxicity.

Moreover, size of nanoparticles also influences their oral toxicity. In general, the oral toxicity increases with decreasing size. In one of the studies, it was observed that oral toxicity of copper nanoparticles increased with decreasing size. More importantly, larger particles were nontoxic even at higher doses, whereas smaller particles were moderately toxic [31].

Furthermore, employing zebrafish as a model to evaluate the* in vivo* toxicity of different gold and silver nanoparticles in the size range of 3, 10, 50, and 100 nm, the researchers reported that AgNPs produce size dependent mortality, whereas, interestingly but not surprisingly, the behaviour of Au NPs was independent of size [31]. Moreover, in concordance with this study, a similar correlation was observed for the large-sized cyanoacrylate nanoparticles, in which toxicity was dependent on the chemical properties and molecular chain length and was independent of particle size [32]; however vice-versa was true in case of small-sized polyacrylate nanoparticles, wherein toxic manifestations were independent of chemical chemistries.

It implies that although size and surface area are important factors in determining toxicity of nanoparticles other factors such as chemical nature of the constituents may also contribute to the intrinsic toxicity of the nanoparticles.

### 2.2. Effect of Particle Shape and Aspect Ratio

**Figure 1:**
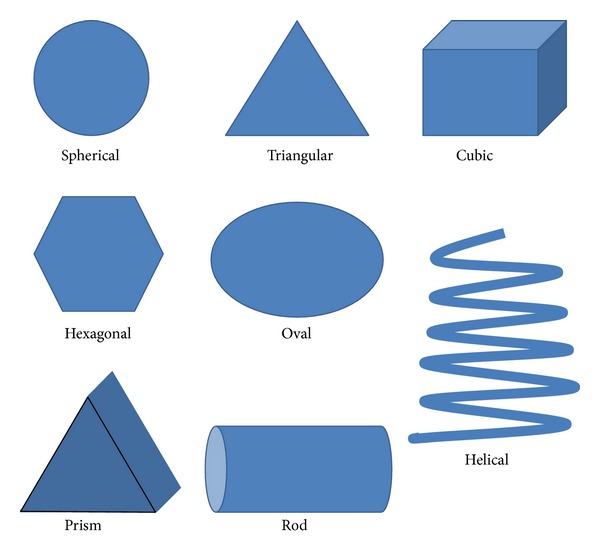
Various shapes of nanoparticles.

There has been flurry of major advancement in the understanding of interplay between particle size and shape for development of more efficacious nanomaterial based targeted delivery system; nevertheless, this also reenforces that their untoward effects should also be examined. As well depicted in Figure 1, nanomaterials come in varied shapes including fibres, rings, tubes, spheres, and planes.

Shape dependent toxicity has been reported for myriads of nanoparticles including carbon nanotubes, silica, allotropies, nickel, gold, and titanium nanomaterials [33–36]. Basically, shape dependent nanotoxicity influences the membrane wrapping processes* in vivo* during endocytosis or phagocytosis [37]. It has been observed that endocytosis of spherical nanoparticles is easier and faster as compared to rod shaped or fibre like nanoparticles [38] and more importantly spherical nanoparticles are relatively less toxic irrespective of whether they are homogenous or heterogeneous [39]. Nonspherical nanomaterials are more disposed to flow through capillaries causing other biological consequences [40]. Studies have shown that rod shaped SWCNT can block K^+^ ion channels two to three times more efficiently than spherical carbon fullerenes [41]. Of note, theshape dependent toxicity of silica allotropies is evident by fact that amorphous silica is used as food additive while as crystalline silica is suspected human carcinogen [33]. Similarly, it has been shown that uptake of gold nanorods is slower than spherical nanospheres [35] and uptake of nanorods reaches maximum when aspect ratio approaches unity [42]. It has been observed that TiO_2_ fibres are more cytotoxic than spherical entities [43].

Moreover, it has also been observed that the higher the aspect ratio, the more the toxicity of particle [44]. In case of asbestos induced toxicity, it was observed that asbestos fibres longer than 10 microns caused lung carcinoma while fibres >5 microns caused mesothelioma and fibres >2 microns caused asbestosis [45] as longer fibre will not be effectively cleared from the respiratory tract due to the inability of macrophages to phagocytise them. Hamilton et al. [36] showed that TiO_2_ fibers with a length of 15 mm are highly toxic compared to fibers with a length of 5 mm and initiate an inflammatory response by alveolar macrophages in mice. The toxicity of fibres with long aspect is closely related to their plasma shelf life. The fibres that are sufficiently soluble in lung fluid can disappear in a matter of months, while the insoluble fibers are likely to remain in the lungs indefinitely. It was also observed that long-aspect ratio particles (SWCNTs) produce significant pulmonary toxicity compared to spherical particles [46]. Further, long MWCNTs cause inflammation of the abdominal wall after inta-abdominal instillation, while no inflammatory responses were observed in case of short MWCNT [47]. Accordingly, as the intricacies of these phenomena increasingly unravel, they would certainly help towards implementation of safer nanotechnology based systems.

### 2.3. Effect of Surface Charge

Surface charge also plays an important role in toxicity of nanoparticles as it largely defines their interactions with the biological systems. Various aspects of nanomaterials such as selective adsorption of nanoparticles [48], colloidal behaviour, plasma protein binding [49], blood-brain barrier integrity, and transmembrane permeability are primarily regulated by surface charge of nanoparticles [50]. Of note, positively charged nanoparticles show significant cellular uptake compared to negatively charged and neutral nanoparticles, owing to their enhanced opsonization by the plasma proteins. Moreover, they have also been shown to induce hemolysis and platelet aggregation [51] owing to which causes severe toxicity to the system.

As surface charge is a major determinant of colloidal behaviour, it specifically influences the organism response upon exposure to nanoparticles by changing their shape and size through aggregate or agglomerate formation [48]. For example the toxicity of dendrimers is influenced by surface charge and it has been observed that positively charged PAMAM dendrimers (G4) exhibit time-dependent toxicity toward zebrafish and mice embryos while anionic PAMAM dendrimers display no toxic manifestations [52]. Similarly positively charged Si nanoparticles (Si–NP–NH_2_) have been shown to be more cytotoxic compared to neutral and negatively charged Si nanoparticles which display minimal to no cytotoxicity issues [53]. Pietroiusti et al. found that acid functionalized SWCNTs exhibits marked embryo toxic effect compared to pristine SWCNTs in pregnant mice models [49].

It has also been observed that surface charge of nanoparticles alters blood-brain barrier integrity and transmembrane permeability. In this regard, it was found that the negatively charged NPs in the size range of 50 to 500 nm permeate skin after dermal administration, whereas no such effects were seen for positively charged and neutral particles irrespective of their sizes. Basically, NPs of 50 nm permeate the skin due to the small size and large specific surface area, whereas 500 nm particles permeate the skin because the high number and density of charged groups lead to a high charge concentration that overcomes the skin barrier [54].

As the interactions of NPs with the biological systems are largely influenced by their surface charge, the research fraternities have employed various amendments to shield or modulate their surface characteristics so as to reduce their toxic manifestations, a glimpse of which has been provided in the later part of the paper.

### 2.4. Effect of Composition and Crystalline Structure

Although it has been emphasized that particle size plays significant role in deciding toxicity of nanoparticles, we cannot simply ignore studies exemplifying comparable toxicities for diverse nanoparticles chemistries having the same dimensions. These studies highlight that the composition and crystalline structure of nanoparticles also influence their toxicity issues. In a study by Griffitt et al. [55] using zebrafish, daphnids, and algal species as models of various trophic levels it was observed that nanosilver and nanocopper with their soluble forms caused toxicity in all tested organisms, whereas TiO_2_ of the same dimensions did not cause any toxicity issues [55], thus emphasizing role of compositions in determining the toxicities of NPs.

Crystal structure also influences the toxicity of nanoparticles and it has been observed that rutile TiO_2_ nanoparticles induce oxidative DNA damage, lipid peroxidation, and micronuclei formation in the absence of light, whereas anatase nanoparticles of the same size and chemical composition did not [26]. Besides, nanoparticles can change crystal structure after interaction with water or other dispersion medium. It has been reported that ZnS nanoparticles become more ordered in the presence of water by rearranging their crystal structure and become more close to the structure of a bulk piece of solid ZnS [56], thereby embarking that the solvent also has a role in the manifestations of toxicities displayed by the nanoparticulate systems as detailed later in the text.

### 2.5. Effect of Aggregation and Concentration

The aggregation states of nanoparticles also influence their toxicities. Basically, the aggregation states of NPs depend on size, surface charge, and composition among others. It has been observed that carbon nanotubes are mainly accumulated in liver, spleen, and lungs without manifesting any acute toxicity but induce cytotoxic effects mostly because of accumulation of aggregates for longer periods [57]. Agglomerated carbon nanotubes have more adverse effects than well-dispersed carbon nanotubes and enhance the pulmonary interstitial fibrosis [58]. Moreover, generally, it has been observed that with increase in the concentration of nanoparticles, the toxicity decreases at higher concentration.

### 2.6. Effect of Surface Coating and Surface Roughness

The surface properties of particles have significant role on toxicity of nanoparticles as they play a critical role in determining the outcome of their interaction with the cells and other biological entities. Surface coating can affect the cytotoxic properties of nanoparticles by changing their physicochemical properties such as magnetic, electric, and optical properties and chemical reactivity [17, 59] and can alter the pharmacokinetics, distribution, accumulation, and toxicity of nanoparticles. It has been known that the presence of oxygen, ozone, oxygen radicals and transition metals on nanoparticle surfaces leads to the generation of ROS and the induction of inflammation by these systems [23, 24, 60]; these certainly influence their associated toxicities issues. To this end, more specifically, Fubini et al. [61] have shown that the specific cytotoxicity of silica is strongly associated with the occurrence of surface radicals and reactive oxygen species on their surfaces.

However, on the other side of coin, surface coating could also be employed to reduce the toxicity issues of the nanoparticles. In general, surface coating can mitigate or eliminate the adverse effects of nanoparticles. In particular, proper surface coating can lead to stabilization of nanoparticles as well as elude release of toxic ions from nanomaterials [62].

To this end, surface modifications of NPs employing hydrophilic and flexible polyethylene glycol (e.g., pegylation) and other surfactant copolymers (e.g., poloxamers and polyethylene) have been considerably used by the research fraternity off late in this advancing field of nanotechnology to stabilize nanoparticulate systems in biological milieu. Although PEG imparts long circulatory time to the nanoparticulate systems mainly by stabilising them in biological system, they could not be indiscriminately used and, more importantly, they have to be chosen with caution, as studies have shown that particles coated with lower molecular weight PEG were quickly eliminated from circulation after injection, whereas QDs coated with high molecular weight remained in the blood circulation for longer time [63].

Surface coatings are important for QDs to render them nontoxic as metallic core of QDs is hydrophobic and is composed of heavy toxic metals like cadmium. In general, secondary coating is needed to increase the QD core's durability, prevent ion leaching, and increase water dispersibility [64]. However, care should be takento choose appropriate coating agents, as weaker surface coatings are prone to oxidative or photolytic degradation leading to exposure of the metalloid core, which may be toxic or can pave the way for unforeseen reactions inside the body [65]. Intriguingly, Chen and Gerion [66] developed silanized QDs (QDs coated with silica) embodying attributes of lack of genotoxicity issues owing to their least interaction with proteins and DNA. Moreover, various biocompatible polymers have also been widely used as coating materials for SPIONs to avoid their toxicity issues [67].

Furthermore, in selecting the appropriate coating material, charge of the coating agent should also be considered. As already discussed that the charge of nanoparticles plays important role in influencing their toxic behaviours, on this line, it has been observed that QDs coated with negatively charged serum protein albumin show a higher liver uptake and faster blood clearance relative to the QDs without albumin [68, 69]. Coatings and functionalization can also reduce the* in vivo* toxicity of carbon nanotubes [70]. Moreover, it has also been demonstrated that spherical gold nanoparticles with various surface coatings have been found to be nontoxic to human cells [71, 72].

Furthermore, as the attributes of nanoparticles such as surface roughness, hydrophobicity, and charge of nanoparticles influence the phenomena of cellular uptake of nanoparticles [73], they indeed influence the toxicity associated with nanoparticles. Surface coarseness dictates the strength of nanoparticle-cell interactions and promotes cell adhesion. Pore structure is critical in cell-nanoparticle interactions. It has been demonstrated that size dependent hemolysis effect of mesoporous silica nanoparticles is only observed when the nanoparticles have long range ordered porous structure [74, 75]. De Angelis et al. [75] showed that nanoporous silicon NPs with a pore size of about 2 nm do not have any toxicity in mouse-models with no histological evidence of tissue pathology. Similarly Park et al. [76] observed that luminescent porous silicon nanoparticles did not show any toxicity in animal models.

### 2.7. Effect of Solvents/Media

Medium/solvent conditions have been known to affect particle dispersion and agglomeration state of nanoparticles, which in turn have effect on their particle size, thereby influencing the toxicity associated with nanoparticles. It has been observed that particles of TiO_2_, ZnO, or carbon black have significantly greater size in PBS than in water; moreover, it is also in general consensus that NPs display different diameters in biological milieu [77, 78]. Accordingly, the toxic effects of nanoparticles show variation depending upon the medium composition in which the nanoparticles are suspended; in another way round, the same nanoparticles exhibit different toxic manifestations when dissolved in different mediums [79, 80]. Although, the dispersing agent may improve the physicochemical and solution properties of nanomaterials formulations, they may also adversely affect the toxicity of nanomaterials.

## 3. Conclusion

Nanotechnology is being envisaged as burgeoning field with many potential human health benefits and with rapid upsurge in the field; it becomes increasingly imperative to evaluate the toxicities issues associated with these nanomaterial based products.

While the toxicity of bulk materials is affected mainly by their composition, however, in case of nanomaterials, additional physicochemical properties such as size, surface area, surface chemistry, surface roughness, dispersion medium, and ability to agglomerate play vital role in determining their toxicity. With newer nanomaterials based products being introduced in the market on daily bases, there is urgent need to reduce the knowledge gap between the physicochemical properties and their influence on the manifestation of toxicities issues. This will certainly pave ways towards maneuvering these physicochemical properties for their safer implementation in diverse fields.
